# Microwave Multicomponent Synthesis

**DOI:** 10.3390/molecules14124936

**Published:** 2009-12-01

**Authors:** Helmut M. Hügel

**Affiliations:** School of Applied Sciences, RMIT University, GPO Box 2476V Melbourne, Victoria 3001 Australia; E-Mail: helmut.hugel@rmit.edu.au

**Keywords:** one-pot reactions, synthetic methods, microwave technology, multicomponent reactions

## Abstract

In the manner that very important research is often performed by multidisciplinary research teams, the applications of multicomponent reactions involving the combination of multiple starting materials with different functional groups leading to the higher efficiency and environmentally friendly construction of multifunctional/complex target molecules is growing in importance. This review will explore the advances and advantages in microwave multicomponent synthesis (MMS) that have been achieved over the last five years.

## 1. Introduction

From an environmental and economic perspective it is becoming obvious that the traditional methods of performing chemical synthesis are unsustainable and have to be changed. Multicomponent coupling reactions provide a solution since they are more efficient, cost effective and less wasteful than traditional methods. The achievement of making multiple bonds in a one-pot multicomponent coupling reaction promotes a sustainable synthetic approach to new molecule discovery. Microwave [MW) irradiation facilitates better thermal management of chemical reactions. The rapid MW heat transfer allows reactions to be carried out very much faster compared to conventional heating methods often resulting in increased product yield. Furthermore, the products of temperature sensitive reactions from kinetic or thermodynamic pathways can be selectively tuned and isolated. Since multicomponent reactions often create complete and complex molecular products in a single synthetic step, it is more accurate to describe this modern organic chemistry procedure as microwave multicomponent synthesis (MMS) rather than microwave multicomponent reactions. It also serves as a pathway to generate molecular diversity that combats the commonly costly and time-consuming drug discovery process whereby few novel therapeutics reach the market place. The added experimental benefits of generating complex structures from simple starting materials without engaging protection-deprotection protocols, lengthy product purification procedures improves the synthetic approach/outcomes for future young scientists wishing to contribute products to a more scientifically innovative society.

Reference to chapter 17, Multicomponent Reactions Under Microwave Irradiation Conditions, in Volume 2 of Microwaves in Organic Synthesis [1] edited by Loupy; Kappe’s review Controlled Microwave Heating in Modern Organic Synthesis [2] and the book chapter Microwave-Assisted Multicomponent Reactions for the Synthesis of Heterocycles by Bagley and Lubinu [3] are good entry points for descriptions of multicomponent synthesis utilizing microwave technology.

## 2. The Biginelli Reaction

**Scheme 1:**
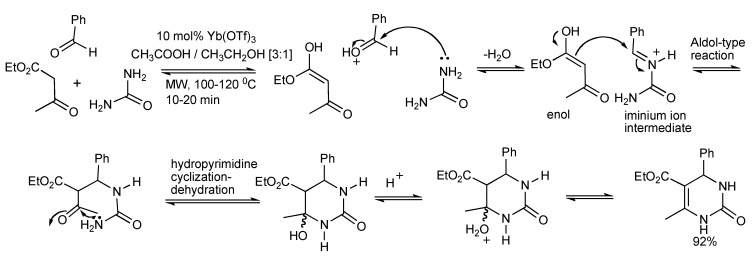
Optimized Biginelli reaction conditions for dihydropyrimidine synthesis.

This is a versatile one-pot synthesis of 3,4-dihydropyrimidin-2(1H)-ones (DHPMs) by the acid catalyzed condensation reaction of three-components: an aromatic aldehyde, a β-ketoester 1,3-dicarbonyl compound and urea (Scheme 1). The optimal experimental conditions [2] with respect to time/temperature of microwave heating, solvent, catalyst type/concentration were achieved using 10 mol% ytterbium triflate catalyst in acetic acid/ethanol (3:1). Microwave heating of the MCR for 10 min at 120 °C produced 92% of isolated DHPM product. Higher temperatures should be avoided, for when the reaction was conducted at 130 °C the yield reduced to 50%. Furthermore a diverse set of the three components coupled with the utilization of robotics enabled the automation of the process that generated library of 48 DHPMs in 12 h.

**Scheme 2:**
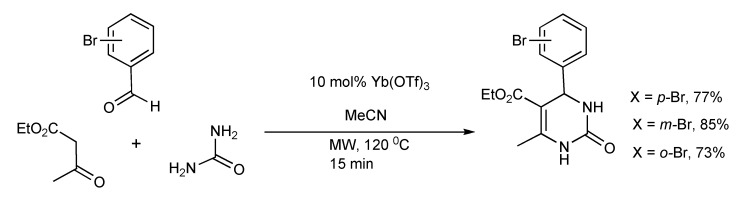
Optimized Biginelli reaction conditions for bromophenyl-substituted DHPM synthesis.

The Biginelli reaction protocol (Scheme 2) was adapted [4] to yield bromophenyl-substituted derivatives of DHPMS on a 1 and 40 mmol reaction scale using single- and multimode microwave reactors respectively. The same experimental conditions of reaction time and temperature were applicable to both microwave reactors, producing similar product yields.

**Scheme 3:**
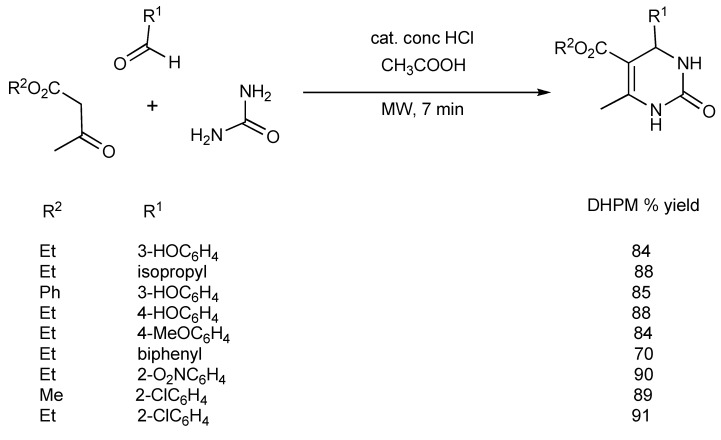
Bronsted acid catalyzed Biginelli DHPM synthesis.

Within a domestic microwave oven, the synthesis of a number of 4-aryl-3,4-dihydropyrimidinones (Scheme 3) has been reported [5] illustrating that both Lewis (see above) and Bronsted acids can be effective catalysts in the Biginelli DHPM reaction.

The modification of the lanthanum chloride Biginelli catalyst by impregnation of LaCl_3_ on graphite support [6] reduced the reaction time for DHPM thione formation from 5 h for conventional heating in ethanol to 8 min using microwave irradiation.

**Scheme 4:**
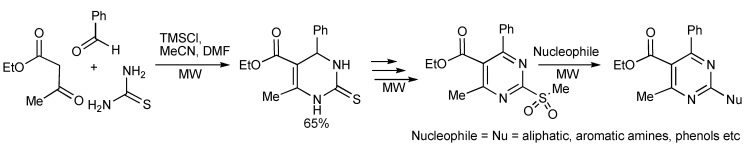
Biginelli reaction for synthesis of 2-amino-4-arylpyrimidines.

A high speed microwave method using the Biginelli MMS has been utilized to prepare the 2-amino-4-(het)aryl-pyrimidine structural motif found in important pharmaceuticals [7]. Employing TMSCl as the inexpensive mediator/catalyst of the reaction with microwave heating, (120 °C, 10 min) a 65% yield of dihydropyrimidine-2-thione was obtained (Scheme 4) which was elaborated into 2-amino-4-arylpyrimidines.

**Scheme 5:**
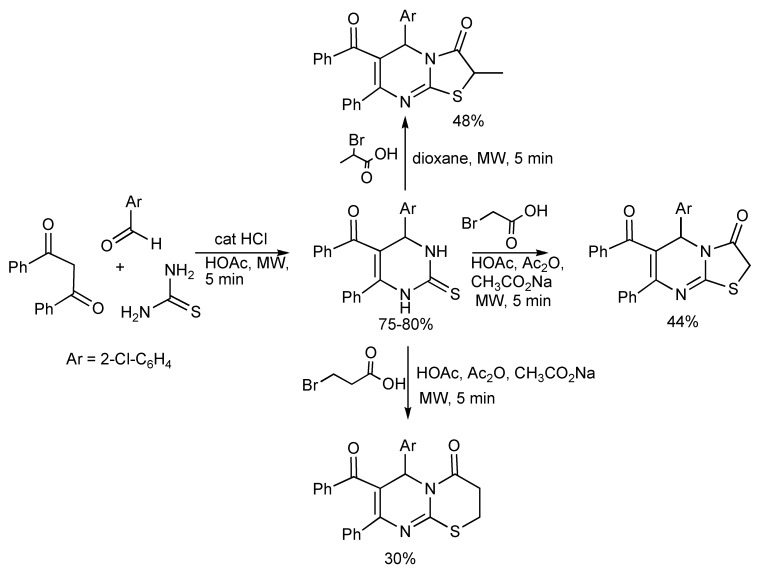
Biginelli reaction to 2-thioxopyrimidines and derivatives.

Two 2-thioxopyrimidines derivatives [Ar = Ph, 2-Cl-C_6_H_4_] were prepared by the Biginelli reaction protocol (Scheme 5). Thus the 5 min MW irradiation of a mixture of 1,3-diphenyl-1,3-propanedione, aryl aldehyde and thiourea in glacial acetic acid plus a few drops of concentrated hydrochloric acid gave the products in 75%–80% yields [8]. The 2-thione DHPMs were transformed into thiazolopyrimidines and pyrimido thiazine derivatives with bromo acids and MW irradiation. When compared to conventional heating, the MW technology completed the two step synthesis much faster [10 min *vs.* 10 h].

**Scheme 6:**
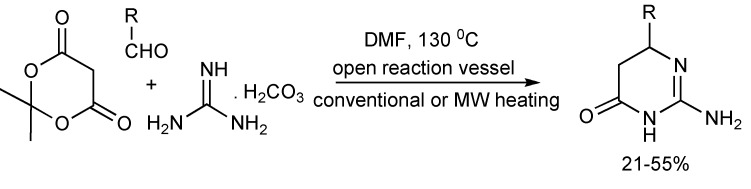
The Biginelli reaction route to 2-amino-DHPMs.

A simple synthesis of 2-amino-DHPMs was achieved when Meldrum’s acid, aliphatic or aromatic aldehydes and guanidine carbonate in DMF were heated to 120–130 °C for 20–25 min (Scheme 6). Similar isolated product yields (21%–55%) were obtained from both conventional and microwave heating. The CO_2_ produced during the reaction increases the internal pressure in sealed microwave reaction vessels, so for safety reasons, this reaction is preferably performed in open vessels [9].

**Scheme 7:**
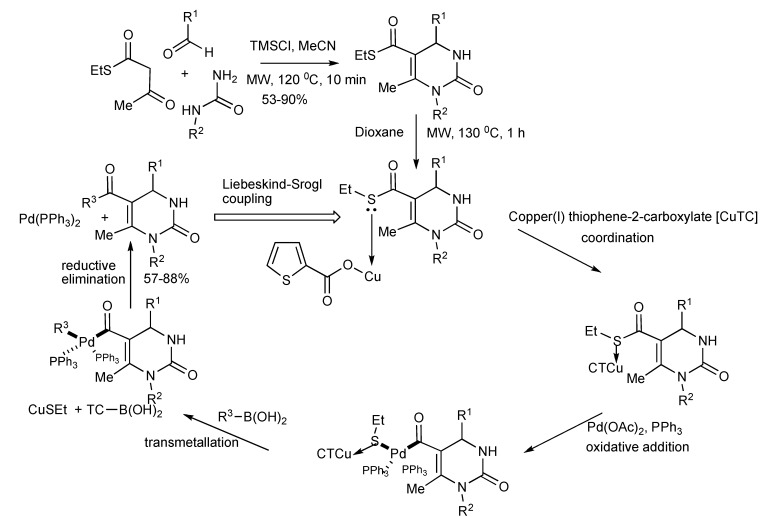
Generation of 5-aroyl-3,4-dihydropyrimidin-2-one library from Biginelli and Liebeskind-Srogl coupling reactions.

Judicious chemical functionalization of the Biginelli MCR substrates expanded the obtainable product molecular diversity featuring the 3,4-dihydropyrimidin-2(1H)-one scaffold [10]. The Biginelli derived DHPM-5-carboxylic acid thiol ester intermediates were converted into DHPM-5-ketone derivatives (Scheme 7) *via* Pd-catalyzed carbon-carbon cross-coupling with boronic acids [Liebeskind-Srogl Coupling] to generate library of 5-aroyl-DHPMs.

In a recent tabulation of the 342 Biginelli catalysts and conditions [11] that have been published, 45 (13%) of these methods utilized microwave irradiation to speed up the reaction rate. As the reaction consists of polar reactants and products, involves polar/charged intermediates and as the ethanol/acetic acid solvent systems are efficient microwave absorbers, should all Biginelli reactions be performed using microwave energy? Since microwave dielectric heating reduces chemical reaction times form hours to minutes, diminishes side reaction products, increases product yields, improves reaction reproducibility and overall chemical synthesis efficiency, the answer is in the positive (more parallel studies of conventional heating/microwave irradiation of MCR are required). For the Biginelli reaction, microwave heating has considerably improved product yields and should be used/considered wherever/whenever possible.

**Scheme 8:**
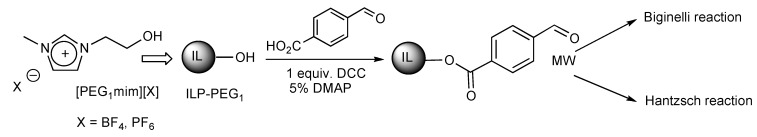
The utilization of IL-PEG technology for the Biginelli, Hantzsch reactions.

Ionic liquid (IL) phase organic synthesis methodology [12] with microwave heating has been successfully applied to the synthesis of Biginelli DHPM, Hantzsch 1,4-dihydropyridines and related compounds. The IL-phase bound aldehyde (Scheme 8) obtained from the coupling of methylimidazolium-ethyleneglycol tetrafluoroborate or hexafluoroborate with 4-formylbenzoic acid was employed. The major advantage of ionic liquid phase technology is that their solubility depends on the selection of the cations and anions employed, allowing phase separation from organic or aqueous phases leading to simple product purification without the use of solvents.

**Scheme 9:**
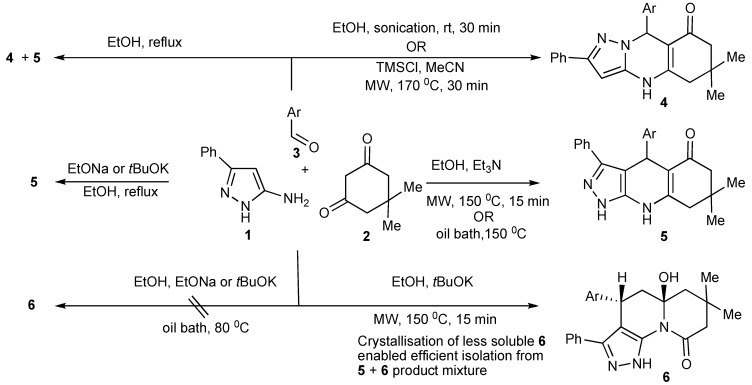
MW-tuning chemo- and regio-selectivity of three-component condensation products.

The product outcomes of MMS performed in protic solvents with substrates having multiple nonequivalent nucleophilic reaction sites allows the tuning of the experimental conditions leading to product control. Thus, the experimental control of the three-component condensation of 5-amino-3-phenylpyrazoles (1), dimedone (2), and aromatic aldehydes (3) selectively formed three tricyclic heterocyclic products, **4**, **5**, **6** (Scheme 9) [13].

**Scheme 10:**
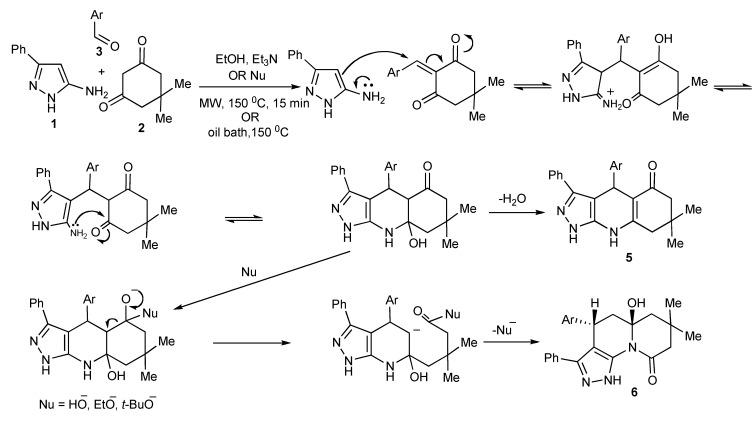
Formation of pyrazoloquinazolinone **5** and pyrazoquinolizinone **6** by three-component-base condensation reactions.

The investigation and optimization of microwave/conventional heating and reagent/reaction conditions of this three-component condensation reaction established that:

- condensation of 5-amino-3-phenylpyrazole **1**, dimedone **2**, and aromatic aldehydes **3** in refluxing ethanol, usually resulted in mixtures of the Biginelli-type DHPMs viz pyrazoloquinolinones **4** and the Hantzsch-type dihydropyridines viz pyrazoloquinazolinones **5** being formed;
- sonication of equimolar mixtures of the three components in ethanol at room temperature produced the Biginelli-type pyrazoloquinolinones **4** in reasonable yields (51%–70%);
- the addition of catalytic amounts of hydrochloric acid to equimolar mixtures of the three components with 15–40 min microwave irradiation at 150–170 °C gave mixtures of **4** and **5**. However using a 2:2:1molar ratio of **1**, **2**, **3**, favoured **4**/**5** product formation in 4/1 ratio (Scheme 9);
- after the addition of trimethylsilylchloride as a reaction mediator in acetonitrile with microwave irradiation for 30 min at 170 °C resulted in almost exclusive formation of the Biginelli type product **4** (>75%);
- condensation of the three-components with triethylamine in ethanol at 150 °C with 15 min of microwave irradiation (76%) and/or oil bath heating at 150 °C (74%) produced the pyrazoloquinazolinones **5** as the single product indicating that this selectivity is due to a thermal effect;
- other experiments that were performed with stronger nucleophilic bases, sodium ethoxide or potassium *tert-*butoxide in ethanol with microwave irradiation at 150 °C, 15 min gave products (38%–75%) that could only be rationalized as involving nucleophilic attack-ring opening and lactam recyclisation to a pyrazoloquinolizinone **6** (Scheme 10) [14];
- condensation of the three-components with sodium ethoxide or potassium *tert-*butoxide in refluxing ethanol resulted in the very much slower formation [when compared to using triethylamine] of only the Hantzsch-type dihydropyridine **5**, indicative that higher reaction temperatures are necessary for this reaction;
- utilization of nucleophilic bases with microwave irradiation gave mixtures of products **5** and **6** with the appreciable differences in the product solubilities simplifying product isolation of compound **6**.

The most influential factors that controlled/guided the product pathways in this multicomponent reaction were the relative substrate concentrations used and the reaction temperature coupled with choice of catalyst. The selective use of conventional and microwave heating provided experimental flexibility.

**Scheme 11:**
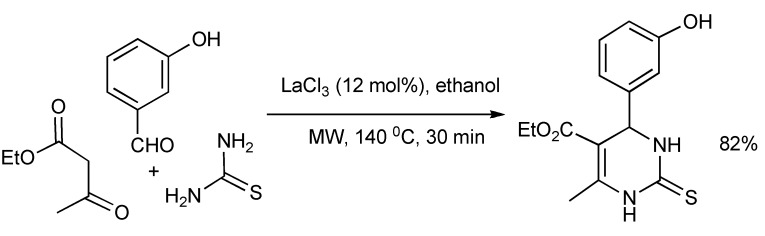
Optimized Biginelli-MMS of Monastrol.

A statistical design of experiment protocol [15] requiring 29 experiments, delivered the optimized reaction parameters of solvent (ethanol), catalyst type/concentration (LaCl_3_/12 mol%), microwave reaction time (30 min) and temperature (140 °C) for the Biginelli MMS of the mitotic kinesin Eg5 inhibitor Monastrol in 82% yield in racemic form (Scheme 11). This is a significant yield improvement as Biginelli reactions typically give low yields.

**Scheme 12:**
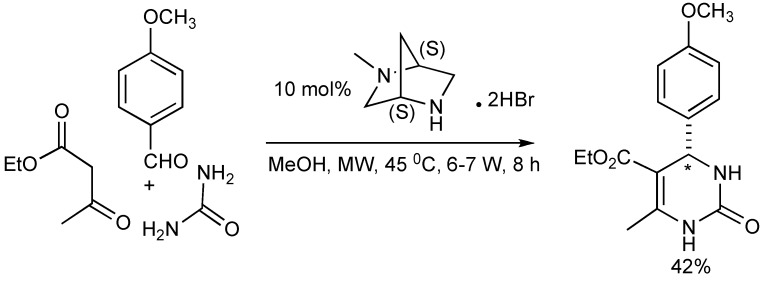
The asymmetric Biginelli-MMS.

The enantioselective Biginelli-MMS (Scheme 12) has been explored [16] with limited success. Using the chiral bicyclic diamine: (1S, 4S)-2-methyl-2,5-diazabicylcol[2.2.1]heptane.2HBr with mild microwave irradiation resulted in 42% yield and 27% ee.

## 3. The Ugi Reaction

**Scheme 13:**
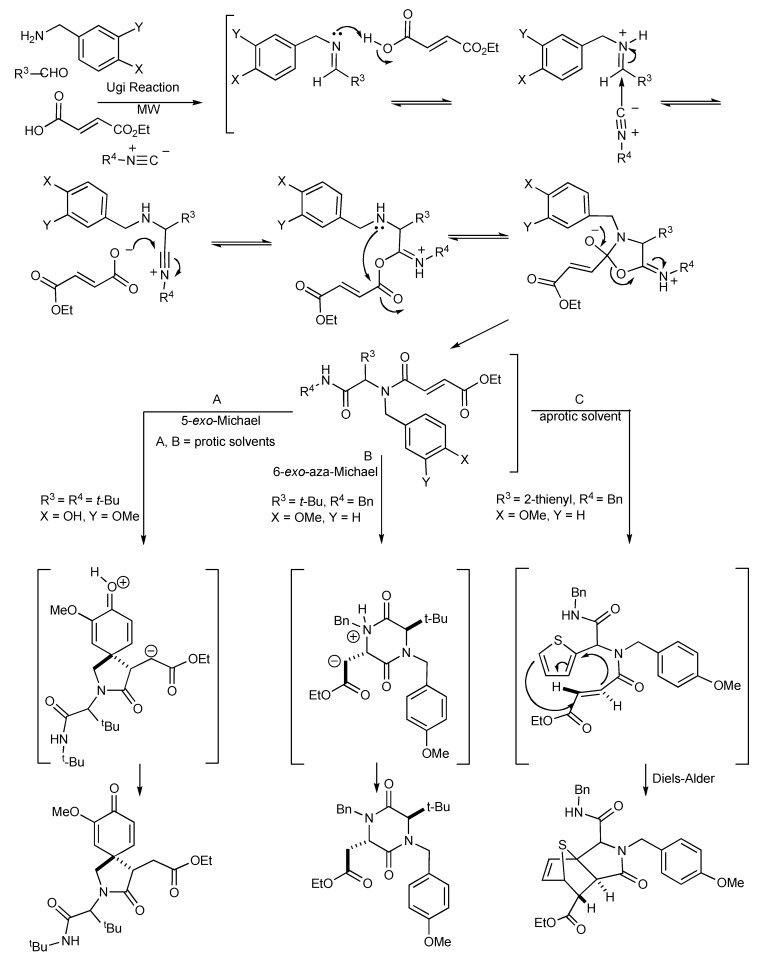
The Ugi reaction: The effects of MW, solvents, bifunctional substrates.

This transformation occurs when isocyanides participate in four-component condensation reactions with an amine, aldehyde or ketone and a nucleophile such as a carboxylic acid, yielding α-acylaminoamides as the Ugi product, as shown in Scheme 13. The impact and advantages of microwave technology on the Ugi four-component coupling products [17] are also illustrated in Scheme 13.

This reaction illustrates the synergies between microwave heating, polar reaction intermediates and the important supporting role of solvents as microwave absorbers in stabilizing intermediates contribute to the bond making process. The resultant molecular product diversity from this MMS are controlled/influenced by microwave irradiation, solvent effects, the bifunctional reactivity of the substrates and the electron donating groups present. In protic solvents, the Ugi product undergoes a 6-exo-aza-Michael reaction to give good yields of 2,5-diketopiperazines (pathway B). Alternatively, the influence of the electron donation/participation of a *para*-OH group initiated an internal 5-exo-Michael reaction leading to the azaspiro-diendione products (pathway A). The zwitterionic nature of the Michael reaction intermediates makes them both microwave active and so these reactions are enhanced by microwave heating.

**Scheme 14:**
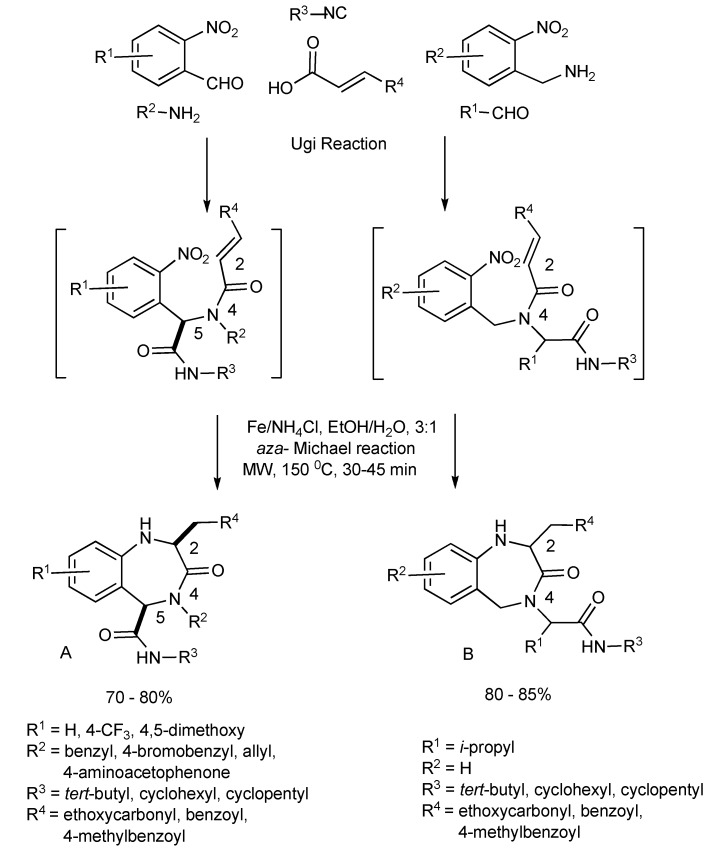
Ugi MMS to racemic 1,4-benzodiazepin-3-ones A or B.

When the reaction mixture was irradiated in dichloromethane, an intramolecular thiophene-based Diels-Alder concerted reaction occurred that resulted in the isolation of a tricyclic lactam adduct (pathway C). It is significant to note that the reaction pathways, A, B and C leading to the molecular products/scaffolds could only be generated with the use of MW heating.

The functional/regiochemical flexibility of the aldehyde and amine (aryl or aliphatic respectively) with the *ortho-*nitro functional group placement in the arene ring of the four-component Ugi condensation reaction product has been further utilized by reduction with Fe(0) and NH_4_Cl followed by an aza-Michael cyclization to produce differentiated 1,4-benzodiazepin-3-ones (Scheme 14) in a one-pot, two step process [18]. The cyclization reaction did not occur without the application of MW irradiation.

Key points:

- the one-pot reaction sequence involves Ugi condensation, nitroarene reduction with Fe(0)/NH_4_Cl and aza-Michael cyclization to benzodiazepines.
- optimal reaction yields were obtained using MW irradiation.
- the microwave intensity controls product selectivity. At high MW intensity (300W, >180 °C, >12 bar, 5–10 min) the Ugi product undergoes a 6-*exo* aza-Michael cyclization faster than the nitro group reduction resulting in formation of 2,5-diketopiperazines [similar to the diketopiperazines formed in Scheme 13]. Lower intensity MW (300W, <150 °C, <10 bar, 30–45 min) exclusively gave the benzodiazepine products.

**Scheme 15:**
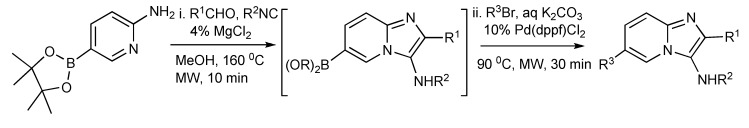
Sequential Ugi plus Suzuki coupling to form 2,3,6-trisubstituted imidazo(1,2-*a*)pyridines.

The intermediate Ugi condensation product derived from 2-aminopyridine-5-boronic acid pinacol ester (Scheme 15) was transformed into 3-amino-imidazopyridines by a MW assisted four-component coupling in a one-pot reaction [19]. The attempted one-pot reaction containing a mixture of MgCl_2_ (Ugi Lewis catalyst) and Pd(dppf)Cl_2_ (Suzuki catalyst) failed.

**Scheme 16:**
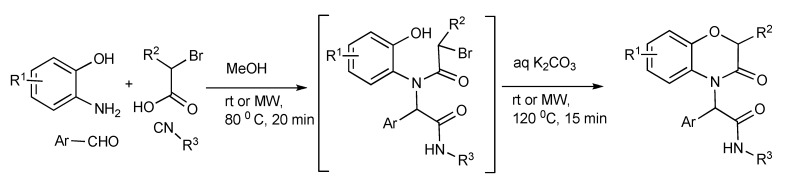
Ugi-MMS with intramolecular O-alkylation furnishing substituted benzoxazines.

The Ugi-four-component reaction plus an intramolecular O-alkylation was performed in a one-pot reaction illustrated in Scheme 16 enabled access to functionalized 3,4-dihydro-3-oxo-2*H*-1,4-benzoxazine products [20]. The optimized MW heating conditions presented in Scheme 16 gave 52%–90% product yields were similar or marginally lower than the room temperature run reactions. However in terms of reaction time efficiency, productivity was significantly higher for the MMS protocol: 35 min (MW) compared to 32–168 h (room temperature) for every reaction.

**Scheme 17:**
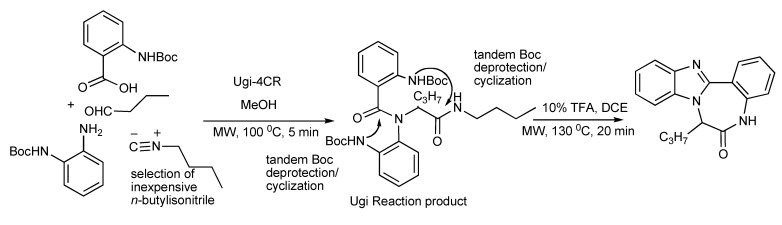
Ugi-MMS with tandem deprotection/intramolecular *N*-cyclizations to triazadibenzoazulenones.

The presence of two Boc protected amino nucleophiles in the Ugi reaction product (63%) upon treatment with trifluoroacetic acid and microwave irradiation enabled a tandem Boc deprotection/cyclization process to proceed *via* a benzimidazole intermediate and a subsequent ring closure to provide triazadibenzoazulenones (70%) (Scheme 17). Control over the order of ring formation was necessary and the optimization of microwave energy input was an important feature in both reactions.

**Scheme 18:**
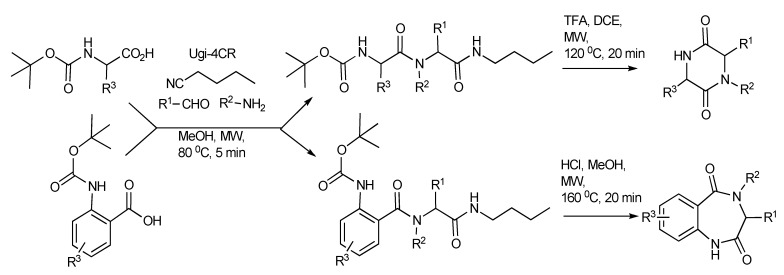
The UDC-MMS strategy leading to diketopiperazines and 1,4-benzodiazepine-2,5-diones.

Application of the readily available, cheap and expendable *n*-butylisonitrile including N-Boc-α-amino acids with amines and aldehydes in Ugi-deprotection-cyclization (UDC)-MMS has also been exploited (Scheme 18) to generate a small number of diketopiperazines (Table 1). The deployment of N-Boc-anthranilic acids in the UDC-MMS methodology furnished 1,4-benzodiazepine-2,5-diones (Table 2)[22].

**Table 1:** The Ugi-DC diketopiperazine products and yields (combined 2 steps).

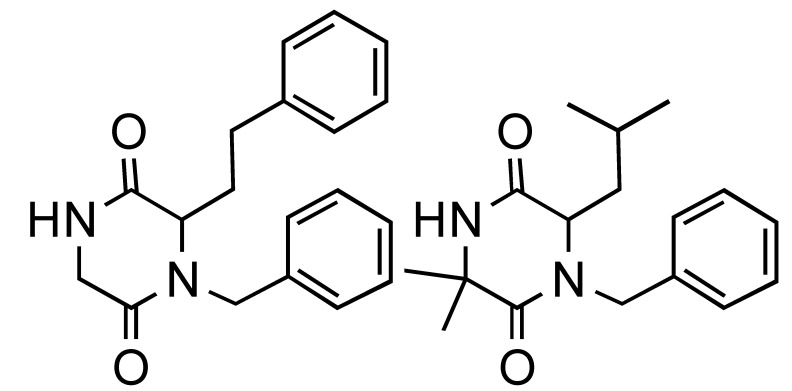		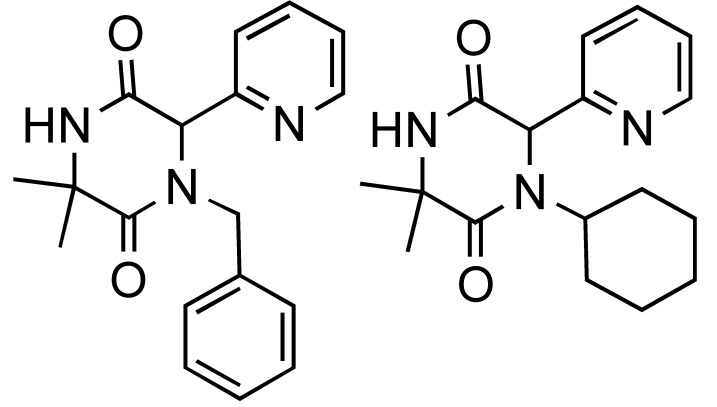		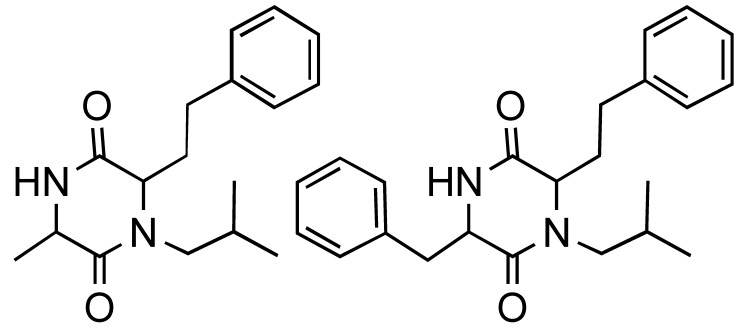	
44%	59%	67%	55%	47%	51%

**Table 2:** The Ugi-DC 1,4-benzodiazepine-2,5-dione products and yields (2 steps).

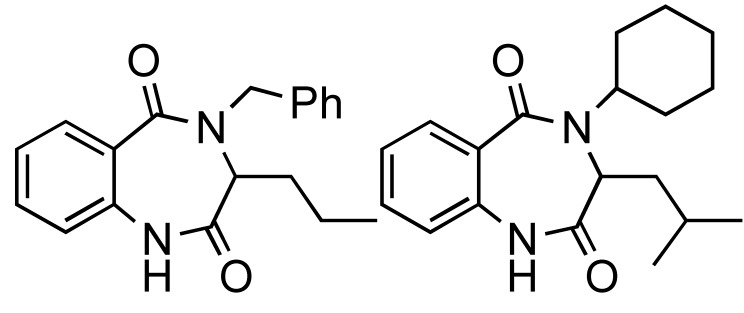		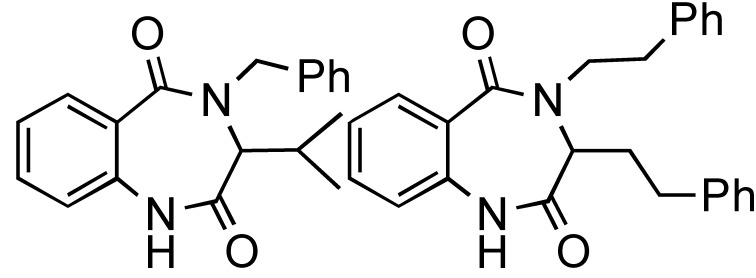		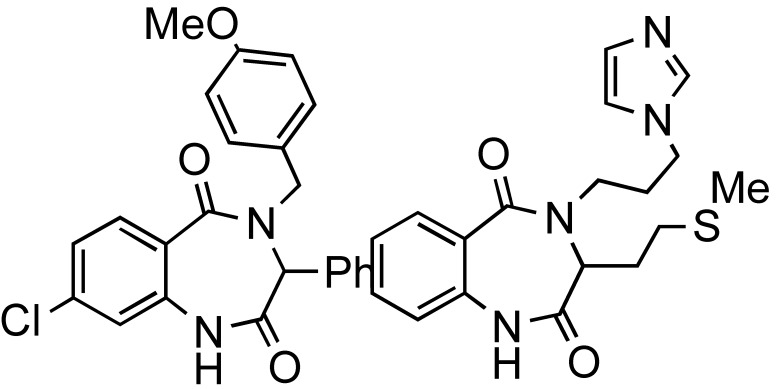	
80%, 75%	91%, 69%	93%, 71%	83%, 60%	50%, 66%	72%, 64%

**Scheme 19:**
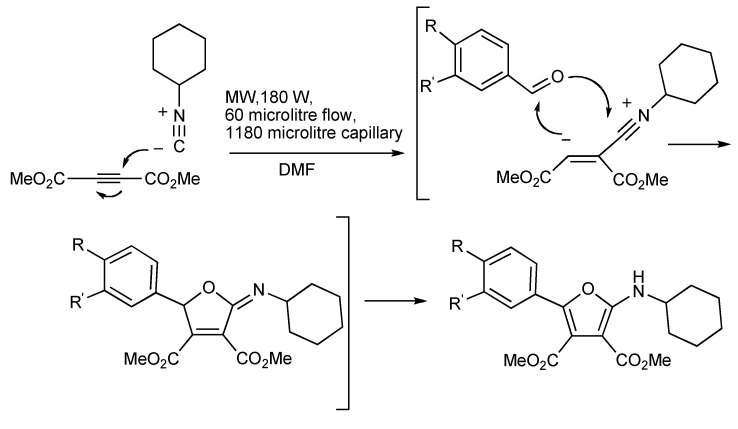
MMS-MACOS provided a small library of 2-amino furans.

The zwitterionic adduct formed by the reaction of cyclohexylisocyanide and dimethylacetylene dicarboxylate is trapped by arylaldehydes producing 2-aminofurans [23]. Conventional conditions required 2–9 h refluxing in benzene and gave modest product yields. The utilization of the microwave-assisted continuous flow (MACOS) procedure produced the tetrasubstituted furans (Scheme 19) in seconds with similar or better yields.

## 4. The Hantzsch Reaction

**Scheme 20:**
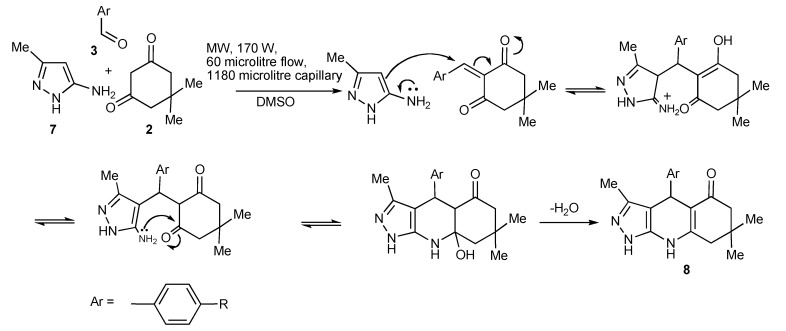
Production of pyrazoloquinolinones by Hantzsch-MMS using MACOS in DMSO.

The one-pot condensation of 1,3-dicarbonyl compounds **2** and/or β-keto esters with an aldehyde **3** and ammonia or amine compounds **7** leading to 1,4-dihydropyridines such as pyrazoloquinolinones **8** is known as the Hantzsch dihydropyridine synthesis (Scheme 20). This synthesis has also been reported [23] to occur speedily using (MACOS) whereby a 1:1:1 stoichiometric ratio of the components is completely mixed albeit in high boiling solvents such as DMF or DMSO to achieve/maximize product yields. The benzaldehyde functional groups used in **3** and the chromatographed product yields **8**, were as follows: R(% isolated yield): -NMe_2_ (94), -CN (55), -CO_2_Me (88), -Br (80), -OH (94), -OMe (71).

**Scheme 21:**
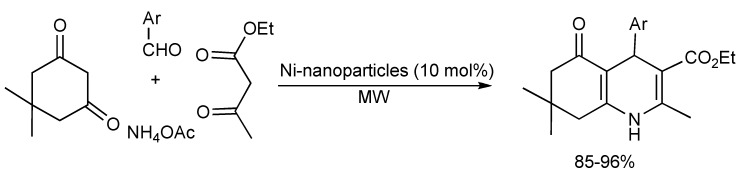
Hantzsch-MMS of polyhydroquinolines catalyzed by Ni-nanoparticles.

A high yielding, solvent free MMS of polyhydroquinoline derivatives employing nanosized Nickel particles as a heterogeneous catalyst prepared *via* the Hantzsch protocol given in Scheme 21 has recently been published [24].

**Scheme 22:**
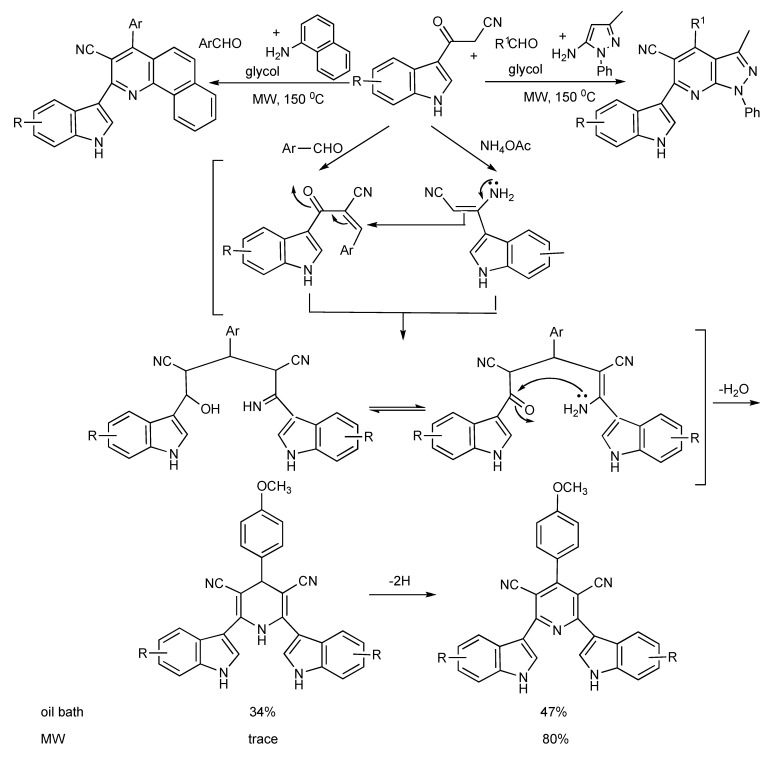
MMS of indolyl-pyridine derivatives.

The application of 3-cyanoacetyl indole in MMS is illustrated in Scheme 22. The MW irradiation at 140 °C of a mixture of 3-cyanoacetyl indole (2 eq.), 4-methoxy benzaldehyde and ammonium acetate in acetic acid-glycol (1:2) produced 4-methoxyphenyl-3,5-dicyano-2,6-di(1H-indol-3-yl)-pyridine in 80% yield, compared to 47% when the reaction mixture was refluxed (oil bath) for 12 h [25]. The versatility of this MMS was illustrated [26] as a 5-aminopyrazole derivative and 2-napthylamine respectively yielded substituted (3’indolyl)pyrazolo[3,4b]pyridine and (3’-indolyl)benzo[*h*]quinoline derivatives (Scheme 22).

**Scheme 23:**
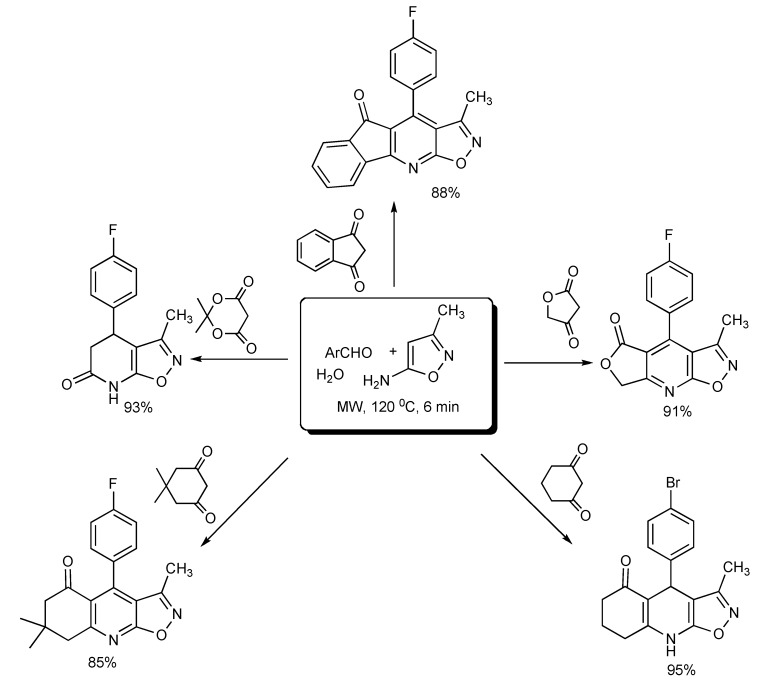
MMS of polycyclic-fused isoxazolpyridines in water.

Water was found to be the most suitable reaction medium and for product isolation for the fast and green MMS of a diverse range of polycyclic-fused isoxazolopyridines [27] as shown in Scheme 23.

**Scheme 24:**
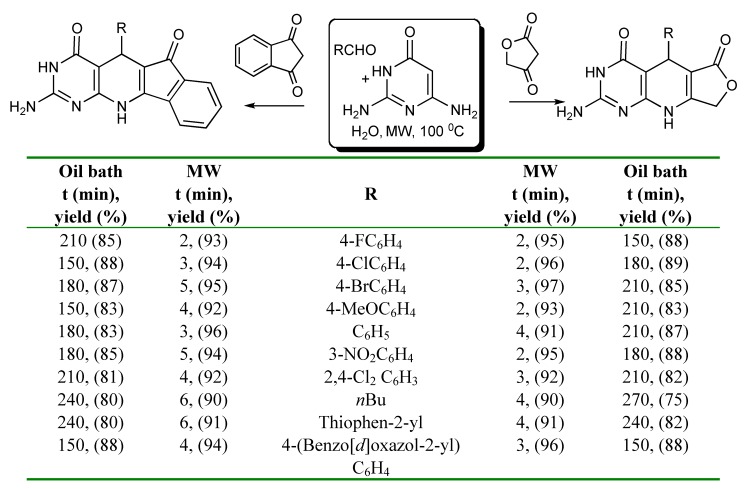
MMS of fuoro- and indeno-pyridopyrimidines in water.

A comparison of microwave and conventional heating conditions for the 3CRs utilising an aldehyde, 2,6-diaminopyrimidine-4(3H)-one and a 1,3-dicarbonyl compound such as tetronic acid or indane-1,3-dione in water showed that the reaction times were much shorter and better yields obtained in the synthesis of fuoro[3’,4’:5,6]pyrido[2,3-*d*]pyrimidines [28] using MW irradiation instead of conventional heating as presented in Scheme 24.

**Scheme 25:**
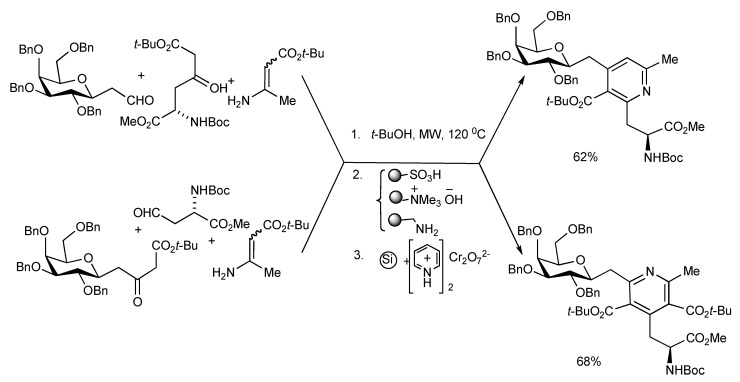
Hantzsch-MMS of 4-(C-galactosylmethyl)-2-(alaninyl)pyridine and 2-(C-galactosylmethyl)-4-(alaninyl)pyridine derivatives.

A number of carbohydrate and amino acid substituted pyridines have been prepared by the MW heating of the aldehyde-ketoester-enamino ester components *via* a Hantzsch cyclocondensation in 58–68% yields. The one-pot Hantzsch three-component reaction mixtures shown in Scheme 25 were irradiated at 120 °C for 1.5 h and the reaction products was treated with three polymer-bound reagents to scavenge unreacted substrates and side products. The diastereomeric dihydropyridines were then oxidised to pyridines with PCC supported on silica gel to give C-galactosylmethyl pyridylalanines that were transformed into pyridine-tethered glycopeptides [29]. In this manner a series of eight pyridine-glycopeptides were synthesized incorporating either gluco or galacto sugars with either the α- or β- configuration at the anomeric carbon.

**Scheme 26:**
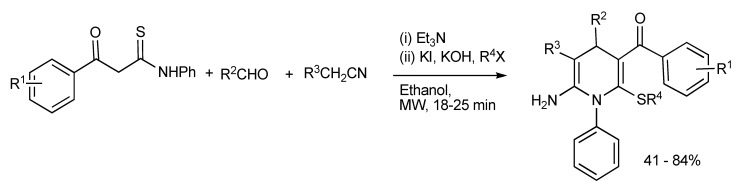
MMS of functionalized hexa-substituted 1,4-dihydropyridines.

A strategic combinatorial method has been developed whereby in a one-pot, two-step multicomponent reaction with readily available substrates such as β-aroylthioamides, predominantly aromatic aldehydes, acetonitrile derivatives plus alkyl halides readily led to the production of highly functionalized hexa-substituted 1,4-dihydropyridine derivatives [30] considered to be complementary to the Hantzsch reaction (Scheme 26).

**Scheme 27:**
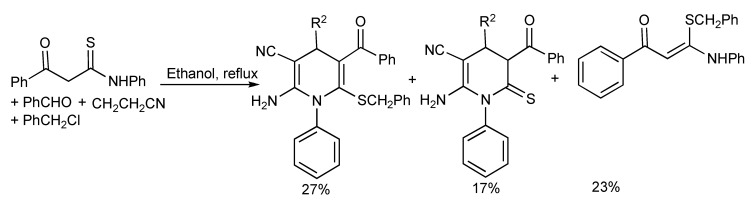
Conventional heating of four-component reaction providing mixtures of hexa-substituted 1,4-dihydropyridines.

Attempts to generate the hexa-substituted 1,4-dihydropyridines *via* a simple one-pot four-component reaction with triethylamine/ethanol under reflux conditions produced a mixture of products (Scheme 27).

**Scheme 28:**
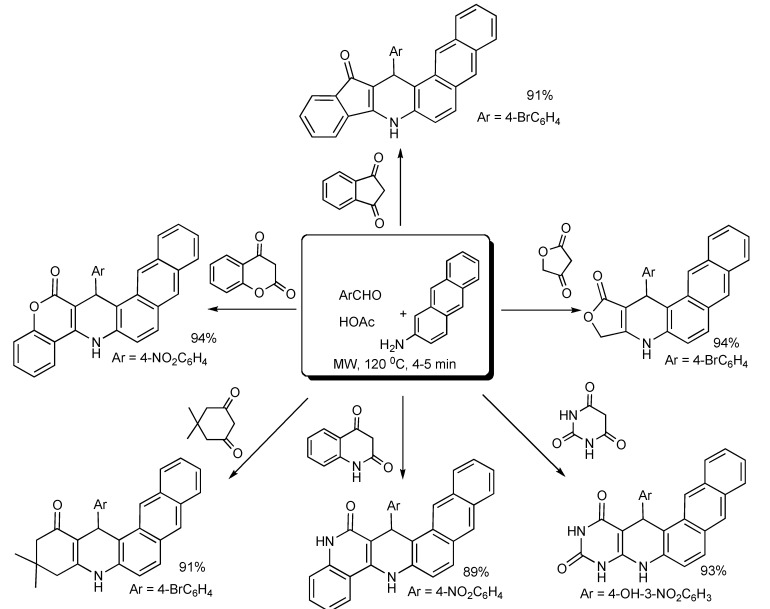
MMS of naphtha[2,3-*f*]quinoline derivatives.

The formation of rigid-ring-framework of azabenz[*a*]anthracene compounds [31] has been achieved *via* the three component combination of equimolar amounts of an aromatic aldehyde, 2-aminoantharacene and a cyclic 1,3-dicarbonyl substrate in acetic acid with MW irradiation (Scheme 28). This protocol offers operational simplicity, small-scale fast synthesis and minimal environmental impact.

**Scheme 29:**
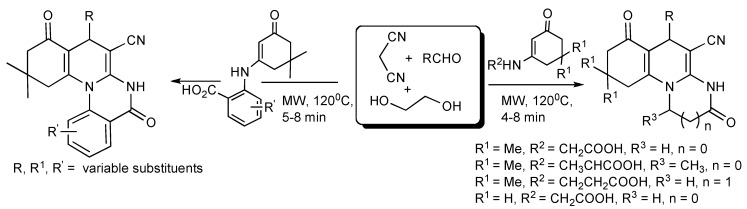
MMS of polysubstituted imidazo[1,2-*a*]-quinazolines, pyrimido[1,2-*a*]-quinolines, quinolino[1,2-*a]*-quinazolines.

MMS has been utilized in developing a high-speed and single-pot combinatorial method for the preparation [32] of a diverse range (42 compounds) of imidazo[1,2-*a*]quinoline, pyrimido[1,2-*a*]quinoline and quinolo[1,2*a*]quinazoline heterocyclic compounds (Scheme 29). The optimized yields/reaction conditions were obtained using ethylene glycol as solvent with MW irradiation at 120 °C.

## 5. Natural Product Synthesis

**Scheme 30:**
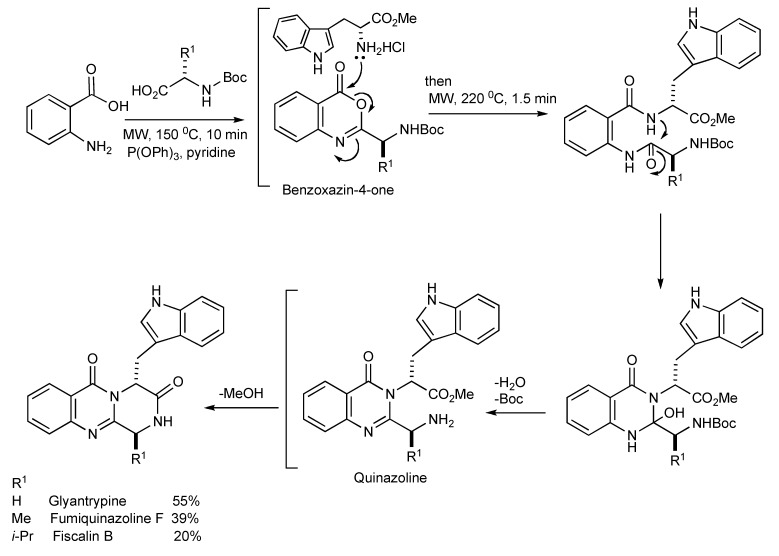
The three-component—MW synthesis of Glyantrypine, Fumiquinazoline F and Fiscalin B.

The alkaloids Glyantrypine, Fumiquinazoline F and Fiscalin B possessing the pyrazino[1,2-*b*]quinazoline-3,6-dione heterocycle have been synthesized [33] by MMS. It is noteworthy that the optimal thermal conditions to give diketopiperazine type cyclization (Scheme 30) required microwave irradiation at 220 °C for 90 seconds.

**Scheme 31:**
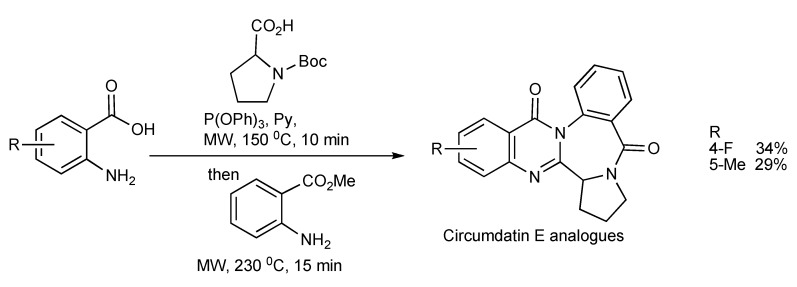
Three-component—MW synthesis of Circumdatin E analogues.

A similar methodology and approach has been applied in the synthesis [34] of Circumdatin E analogues (substituted quinazolinobenzodiazepine alkaloids) as shown in Scheme 31.

**Scheme 32:**
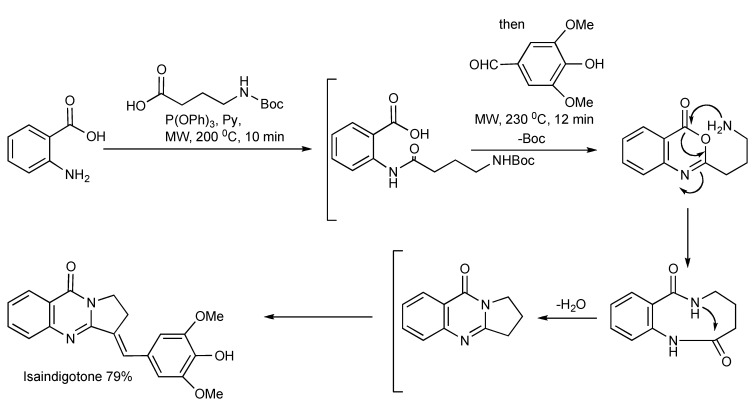
Three-component - MW synthesis of Isaindigotone.

Isaindigotone (Scheme 32) was prepared [35] in a two stage MW-assisted one-pot reaction. Anthranilic acid (1 eq.), 4-(*tert*-butoxycarbonylamino)butyric acid (1 eq.) with P(OPh)_3_ (1.2 eq.), in pyridine solvent was MW irradiated at 200 °C for 10 min, whereupon the introduction of 4-hydroxy-3,5-dimethoxybenzaldehyde (1.2 eq.) and MW irradiation at 230 °C for 12 min gave the product in 79% yield.

**Scheme 33:**
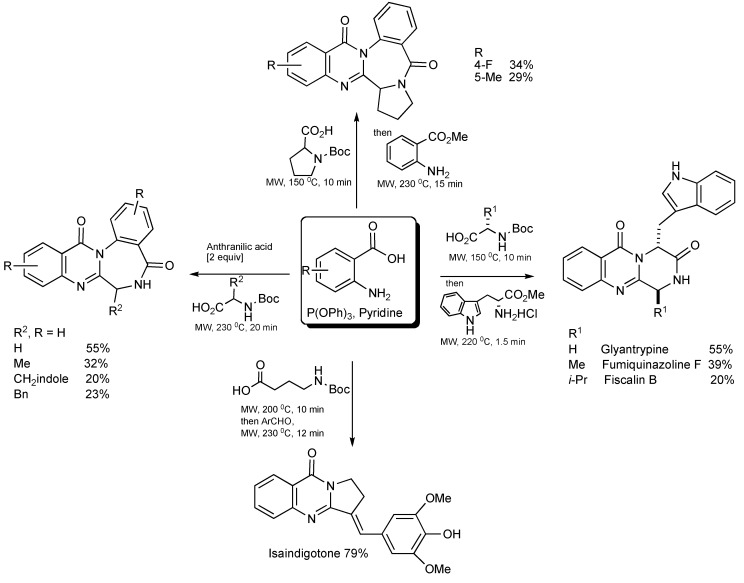
Summary of MMS of the Quinazolin-4-one scaffold.

The focused application of MW technology has enabled the synthesis of products with the quinazoline ring scaffold (Scheme 33). Reaction mixtures requiring only anthranilic acid/derivatives, various NH-Boc protected amino acids, triphenyl phosphine and pyridine generated a range of natural products. This clearly demonstrates the powerful combination of MW and multicomponent synthesis.

**Scheme 34:**
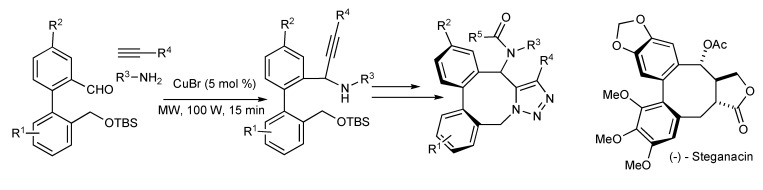
Utilization of MMS in the synthesis of Steganacin aza-analogues.

The benefits of focused microwave irradiation contributed to the efficient multicomponent synthesis of aza-analogues of (-)-Steganacin presented in Scheme 34 [36], a naturally occurring bisbenzocyclooctadiene lignan lactone with antileukemic and tubulin polymerisation inhibitory activity.

**Scheme 35:**
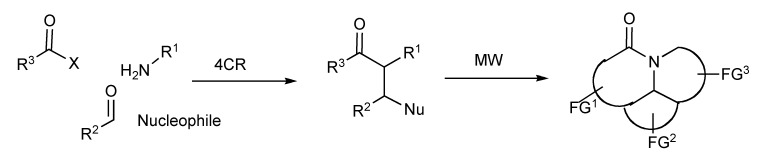
MMS of multiring heterocycles.

Synthetic methods for the efficient generation of families of functionally and stereochemically diverse molecules, particularly those resembling/simplifying structures found in natural products or serving as potential pharmaceuticals have been accessed by multicomponent reactions in tandem with cyclization strategies [37] that utilized MW technology as depicted in Scheme 35.

**Scheme 36:**
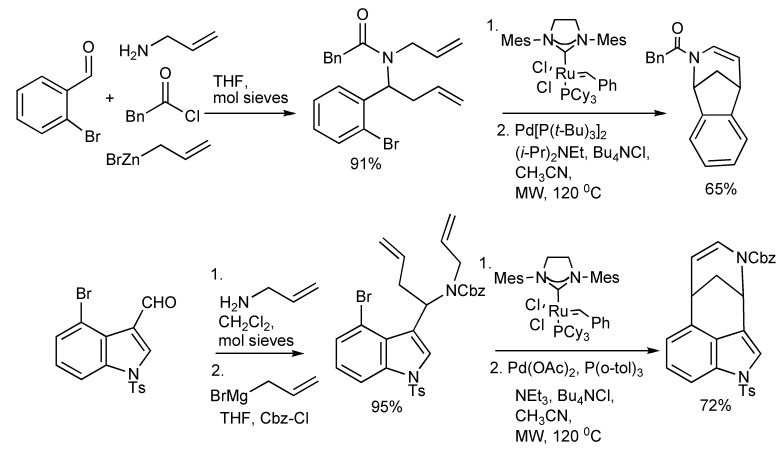
Alkaloid synthesis *via* MMS/RCM/Heck reactions.

Two multicomponent reactions combined with MW irradiation to facilitate ring closing metathesis (RCM) reactions [37] are illustrated in Scheme 36.

## 6. Heterocyclic Synthesis

**Scheme 37:**
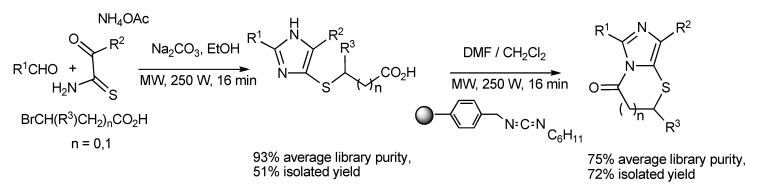
The parallel MMS of imidazothiazol-3-one and imidazothiazin-4-one products.

The use of multimode MW irradiation allowed parallel MW-driven reactions to proceed to generate a diversely substituted library of products quicker than using conventional heating methods [38]. A 24-membered library of substituted 4(5)-sulfanyl-1*H*-imidazoles was produced by MMS in 16 min (Scheme 37). After simple workup, and the utilization of a polymer-supported diimide coupling reagent with parallel MW heating resulted in a second product library composed of imidazo[5,1-*b*]-thiazin-4-ones which was also completed in 16 min.

**Scheme 38:**
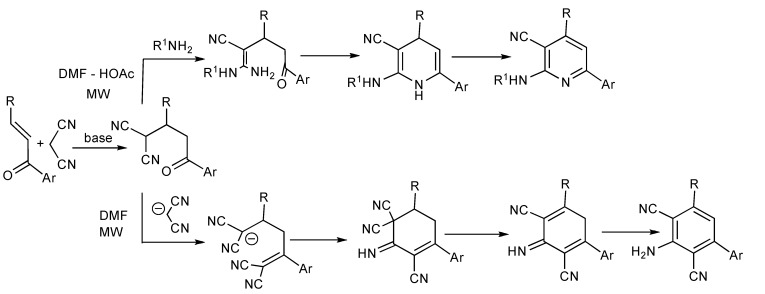
The chemoselective MMS of *N*-substituted 2-aminopyridines and substituted 2,6-dicyanoanilines.

A series of 22 *N*-substituted 2-aminopyridines were prepared [39] in good yields (76%–88%] when a mixture of a chalcone, propanedinitrile and an aliphatic amine in DMF-HOAc [4:1] was irradiated at 100 °C for 4 min. Only 2,6-dicyanoanilines were isolated when the reaction was performed in neat DMF suggesting that the chemoselective synthesis was controlled by the amine basicity and the solvent employed. This implies that the Michael addition product undergoes nucleophilic attack by the amine R^1^NH_2_ followed by condensation/cyclization/aromatization leading to the substituted pyridines (Scheme 38). Alternatively when 2 eq. of propanedinitrile are present in pure DMF, the chalcone undergoes sequential Michael addition and *in situ* Knoevenagel condensation reactions followed by nucleophilic ring closure and aromatization to furnish substituted anilines. Polysubstitued 2,6-dicyanoanilines [40] have previously been prepared by MMS in solution or on polymer support from *in situ* chalcones generated from the corresponding aldehydes and ketones with triethylamine or piperidine.

**Scheme 39:**
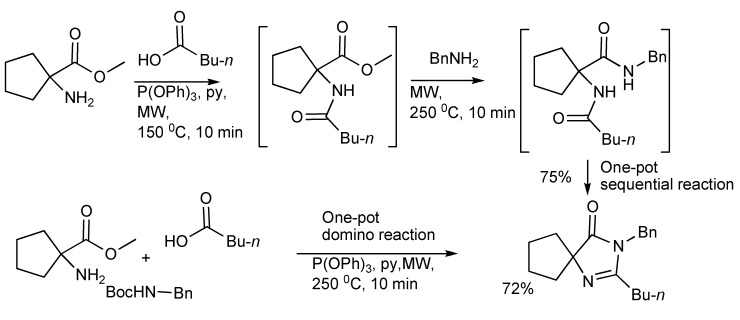
MMS of spiroimidazolinones by one-pot sequential and domino reactions.

**Scheme 40:**
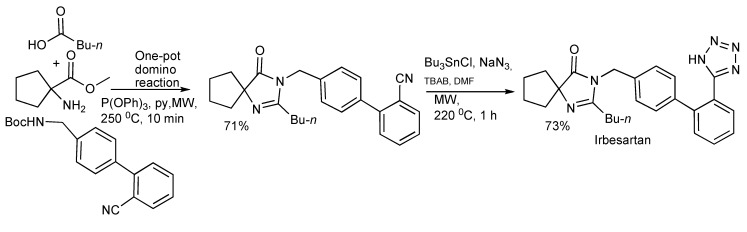
The synthesis of Irbesartan by a one-pot two-step reaction sequence.

The synthesis of spiroimidazolinones [41] has been obtained *via* MMS by the routes of one-pot sequential reactions and one-pot domino reactions (Scheme 39) with both paths having synthetic merit. The ready application and simplicity of this approach was illustrated by the rapid two-step synthesis of the antihypertensive drug Irbesartan outlined in Scheme 40.

**Scheme 41:**
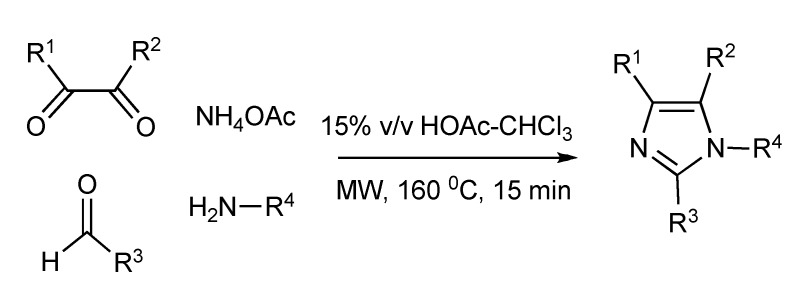
Optimized MMS of imidazoles.

A four-component microwave synthesis [42] was developed and optimized to generate a structurally diverse range [48 member library] of mono-, di-, tri- and tetra-substituted imidazoles (Scheme 41). MW irradiation at 160 °C for 15 min plus using the solvent mixture of acetic acid-chloroform (15%, v/v) achieved the best imidazole yields. The nature of the diketone, especially when R^1^/R^2^ = H gave very low product yields (<10%) limiting the scope of this MMS. Unsymmetrical diketones produced two-regio-isomer imidazole products.

**Scheme 42:**
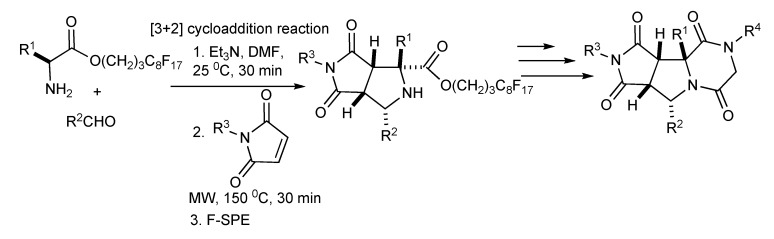
MMS of piperazinedione-fused tricyclic compounds.

A fluorous synthesis was developed [43] involving a three-component one-pot [3+2] cycloaddition of fluorous amino esters, aldehydes and maleimides with MW irradiation yielded bicyclic proline products (Scheme 42) which were transformed by acylation, nucleophilic substitution and lactamization reactions to deliver a 90 member library piperazinedione-fused tricyclic analogues.

**Scheme 43:**
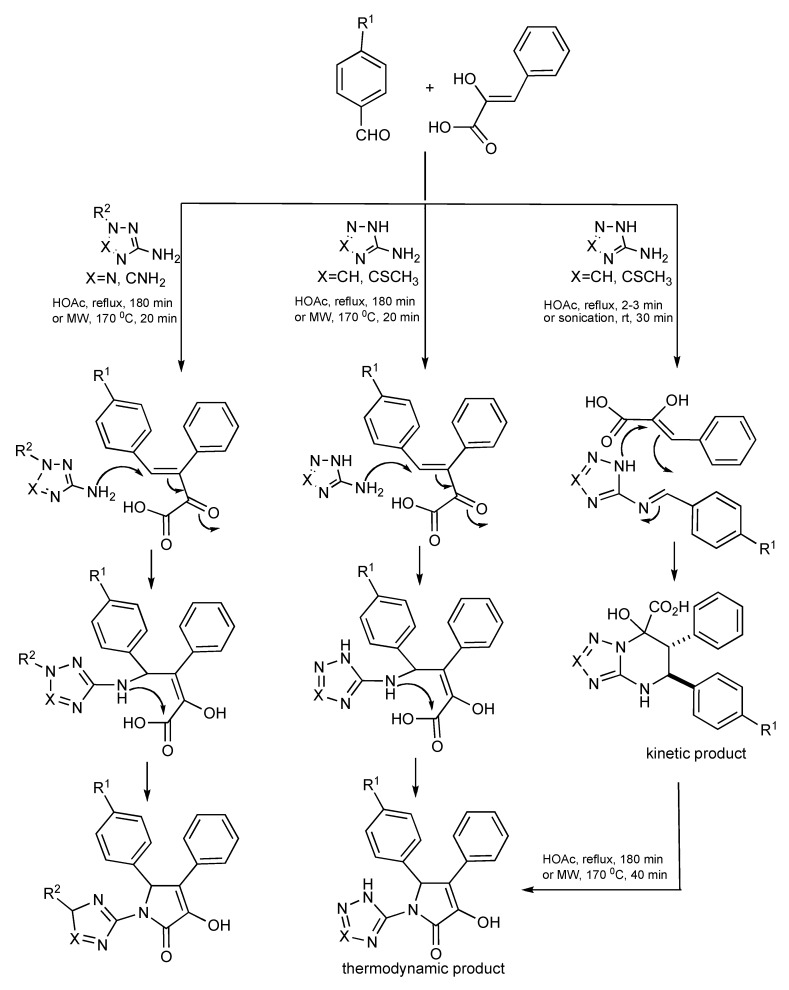
MMS products from aldehydes, phenylpyruvic acid and aminoazoles.

A good example that features how three interacting components [aminoazoles, aldehydes and arylpyruvic acid] can influence the interplay between the chemical reactants and the selected experimental conditions such as conventional, MW heating, sonication, reaction temperature and reaction time have on product outcomes has been published [44]. Room temperature sonication or briefly refluxing aromatic aldehydes with 2-amino triazoles in acetic acid forms azomethines which undergo a heterocyclization reaction with phenylpyruvic acid providing a substituted triazolo[1,5-*a*]pyrimidine-7-carboxylic acid as the kinetic product as shown in Scheme 43. Using extended reflux times (180 minutes) or higher temperatures with MW irradiation suggests that the aldol product formed between the aldehyde and acid then further reacts with either the 2-aminotriazole or 2-aminotetrazole forming the respective thermodynamic pyrrolone products.

**Scheme 44:**
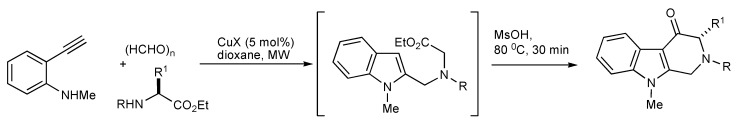
MMS of the 4-oxotetrahydro-β-carboline scaffold.

Upon completion of the formation of the indole intermediate by a copper-catalyzed domino three-component coupling-cyclization involving ethynylaniline, paraformaldehyde, an amino ester in dioxane under MW irradiation, it was treated with MsOH at 80 °C for 30 min to furnish 4-oxotetrahydro-β-carbolines [45] as depicted in Scheme 44.

**Scheme 45:**
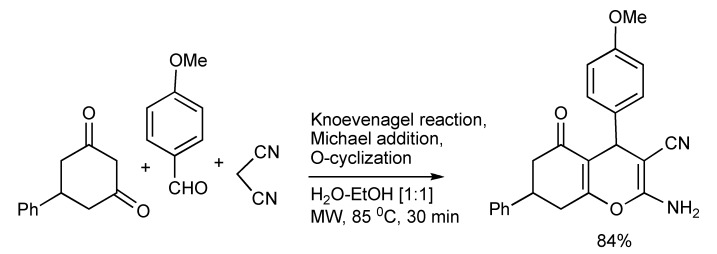
MMS of a substituted 2-aminochromene.

The multicomponent reaction protocol was applied in the synthesis of a 25 member series consisting of four stereoisomers of 4- and 7- substituted 2-amino-5-oxo-5,6,7,8-tetrahydro-4*H*-chromene-3-carbonitriles [46] used in SAR studies and found to inhibit the excitatory amino acid transporter subtype 1. MW heating promoted the production of the analogue shown in Scheme 45.

**Scheme 46:**
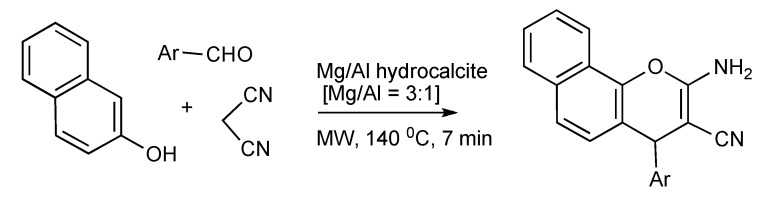
MMS of 2-aminochromenes using Mg/Al catalysis.

Exploitation of the catalytic efficacy of Mg/Al hydrocalcite has enabled the MW, solvent free synthesis of 2-aminochromenes [47] by utilizing the components,1-naphthol, aromatic aldehydes and malononitrile as outlined in Scheme 46.

**Scheme 47:**
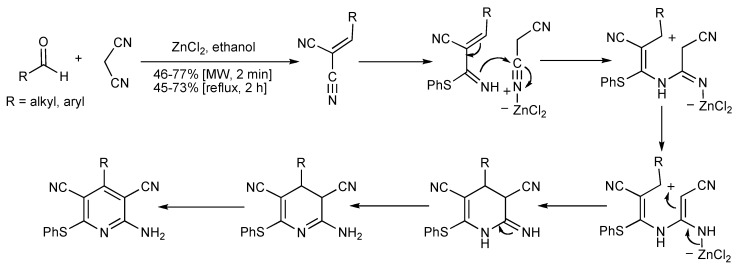
Zinc chloride catalyzed MMS of 2-amino-3,5-dicarbonitrile-6-thio-pyridines.

The ZnCl_2_-catalyzed one-pot 3CR forms 2-amino-3,5-dicarbonitrile-6-thio-pyridines [48] using conventional or MW heating in better yields (45%–77%) (Scheme 47) when compared to reports [49] using base catalysts like DABCO or triethylamine (20%–48%) and conventional heating methods.

**Scheme 48:**
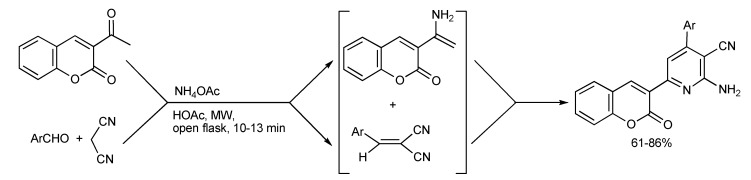
MMS of coumarin substituted pyridine derivatives.

It has been discovered [50] that 3-acetyl coumarin and ammonium acetate forms an enamine that promotes the Michael addition with the Knoevenagel condensation product formed from aromatic aldehydes, malonitrile in acetic acid under MW irradiation. In this manner, a series of 2-amino-6-(2-oxo-2H-chromen-3-yl)-4-pyridine-3-carbonitriles (Scheme 48) were synthesized in better yields and faster by MMS than by conventional heating procedures.

**Scheme 49:**
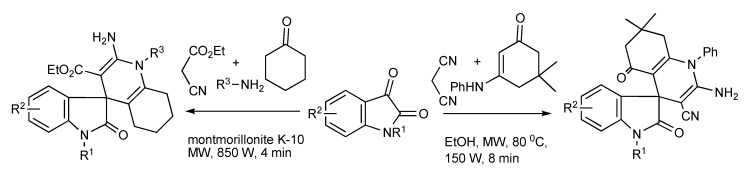
MMS protocols to spirooxindoles.

The MMS method has been employed to efficiently transform isatin derivatives into spirooxindoles. Thus isatins when reacted with malononitirile and enaminones formed spiro[indoline-3,4”-quinoline] derivatives [51]. Alternatively, the utilization of a solvent-free process by condensation of isatins, primary amines, ethyl cyanoacetate and cyclohexanone delivered new spiro-1,4-dihydropyridines [52] as outlined in Scheme 49.

**Scheme 50:**
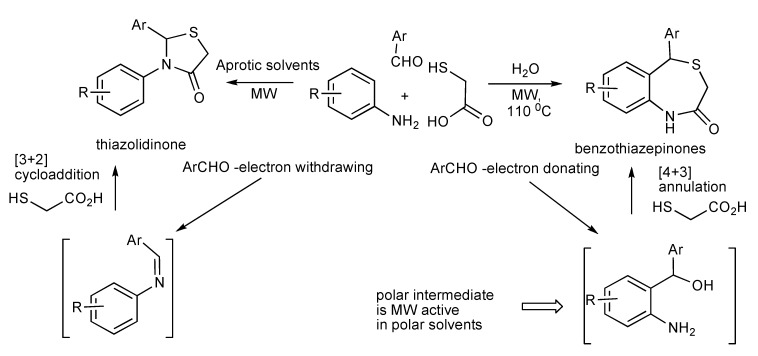
MMS influence by solvent and substituent effects.

Investigations have shown that the MMS product formed between an aromatic aldehyde, aniline and mercaptoacetic acid is controlled by the nature of the solvent and substituents effects of the reaction components [53]. Thus the reaction shown in Scheme 50 in water provided benzothiazepinones and in benzene, dichloromethane, DMF and THF produced thiazolidinones. With aromatic aldehydes containing electron-donating functional groups formation of benzothiazepinones are favoured, whilst electron-withdrawing substituents produced thiazolidinones. However this aldehyde substituent-product chemoselectivity outcome was overridden by the use a very electron-rich amine component such as 3,4-(methylenedioxy)aniline that exclusively formed the benzothiazepinone products.

**Scheme 51:**
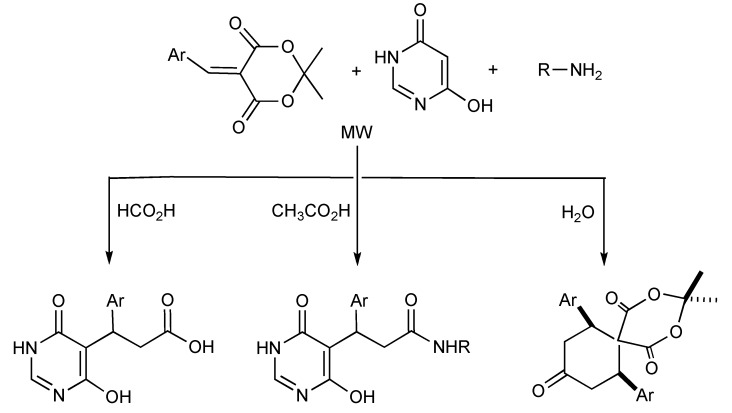
Solvent-selective MMS of 6-hydroxy-3-pyrimidin-5-yl propanoic acid, 6-hydroxy-3-pyrimidin-5-yl propanamide and spiro [5,5]undecane-1,5,9-triones.

One-pot MMS are noted for their high degree of atom transfer efficiency that is conducive for applications in combinatorial chemistry and diversity-oriented synthesis of libraries of compounds for high throughput screening in medicinal chemistry. The development and impact of solvent directed chemoselective reactions [54] in MMS was effectively utilized when arylidene-Meldrum acids, 6-hydroxypyrimidin-4(3*H*)-one and various aliphatic amines were irradiated in various solvents as illustrated in Scheme 51. Aromatic amines only reacted with the arylidene-Meldrum acid component to give quinolin-2(1*H*)-one derivatives. Reaction mechanisms for all the products obtained have been proposed.

**Scheme 52:**
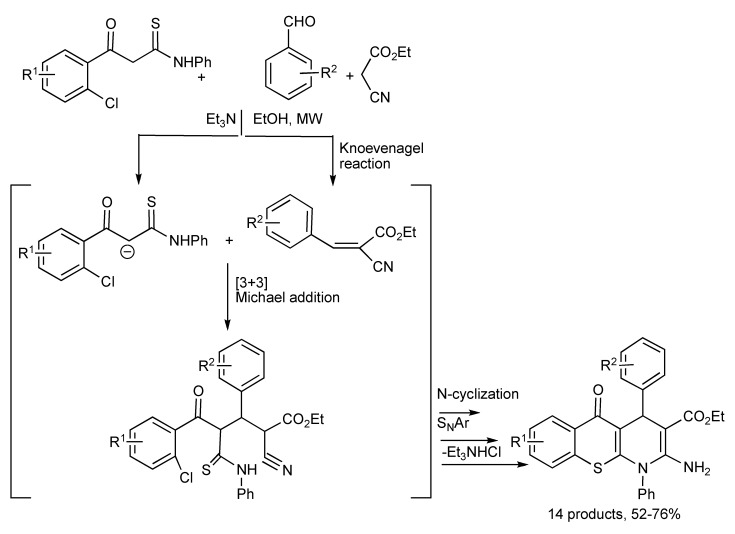
MMS of tricyclic thiochromeno[2,3-*b*]pyridine derivatives.

The facile MMS of fourteen fused tricyclic thiochromeno[2,3-*b*]pyridines [55] was achieved by a one-pot reaction sequence [3+3] Michael addition, N-cyclization and S_N_Ar of β-(2-chloroaroyl) thioacetanilides with aromatic aldehydes and ethyl 2-cyanoacetate as outlined in Scheme 52 below.

**Scheme 53:**
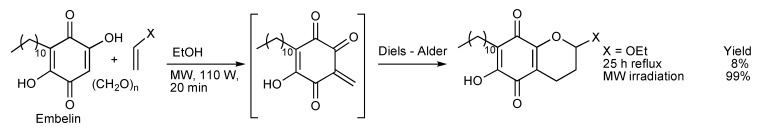
The synthesis of pyrano-1,4-benzoquinone adducts.

The application of MW irradiation significantly enhanced the synthesis yields of pyrano-1,4-benzoquinones [56] obtained by consecutive Knoevenagel and hetero Diels-Alder reactions performed on a mixture of Embelin, paraformaldehyde and ethyl vinyl ether and other dienophiles (Scheme 53).

**Scheme 54:**
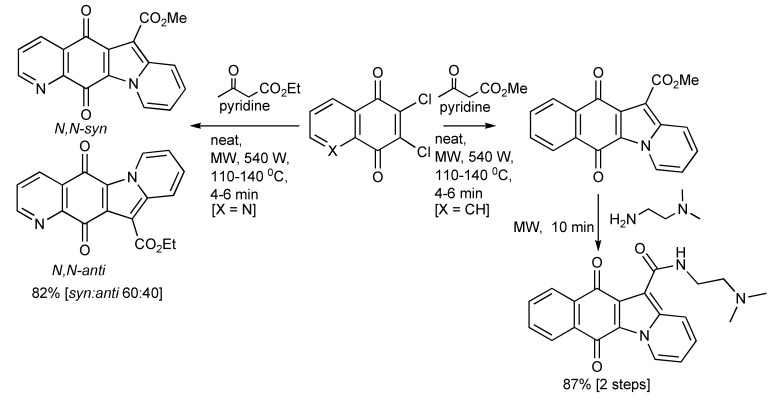
The MMS of heteropolycyclic compounds.

The three-component synthesis of heteropolycyclic compounds including aza-benzofluorenedione and naphthindolizinedione derivatives and related compounds [57] performed with or without solvents was most efficiently achieved using MW heating (Scheme 54). The regioselective production of the *N*,*N-syn* and *N*,*N-anti* tetracyclic aza-compounds was also explored by the presence of solid supports and with catalytic quantities of metal salts. The best product selectivity [*N*,*N-syn:N*,*N-anti* 8:92] were achieved with MgCl_2_.

**Scheme 55:**
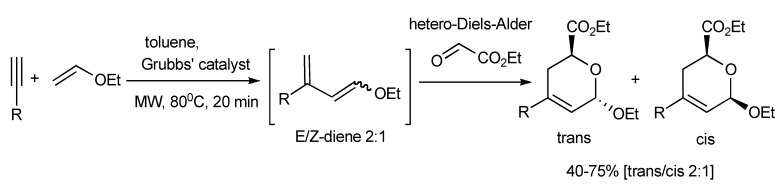
MMS of 2,3-dihydropyrans.

**Scheme 56:**
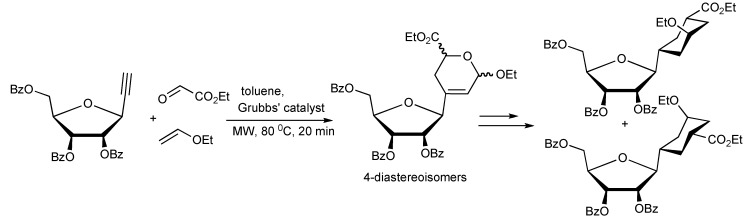
MMS approach to furanose-pyranose 1,3-C-C-linked-disaccharides.

A novel MMS *via* an enyne-cross metathesis-hetero-Diels-Alder reaction facilitated by the 2nd generation Grubbs’ catalyst yielded 2,3-dihydropyrans (Scheme 55) [58]. The trans/cis 2:1 product ratio was rationalized to result from the predominance of the *exo* hetero-Diels-Alder reaction. This MMS protocol was applied in the synthesis furanose-pyranose C-C-linked disaccharides (Scheme 56).

**Scheme 57:**
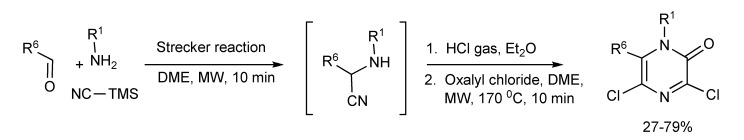
MMS of N-1, C-6-disubstituted 3,5-dichloro-2(1*H*)-pyrazinones.

The α-aminonitrile intermediates from the one-pot, two-step Strecker-MMS were ring closed to produce a range of N-1, C-6-disubstituted 3,5-dichloro-2(1*H*)-pyrazinones presented in Scheme 57 [59]. A weak nucleophile component such as aniline R^1^ = phenyl), gave diminished product yields (27%–29%).

## 7. Conclusions

The use of MW technology in MMS achieves significant laboratory time saving and also often simplifies the experimental reaction requirements enabling the same building blocks to be selectively transformed into different classes of compounds. This is particularly relevant for high-energy heterocyclic reactions. The predominant use of protic solvents, leads to quicker, greener and therefore more environmentally friendlier chemistry. Many examples were presented whereby only the use of MW technology enabled product tuning. A common feature of MMS is the significant influence solvents, substituents and the intensity of MW irradiation have on product formation. Of course overcoming the challenges to perform large scale MMS, the production of not only diversely decorated molecular scaffolds but to design reactions to deliver many specifically targeted compounds as well as finding new multifunctional substrates, will ensure that future innovations will be influential in making MMS an even more useful synthetic method for generating complex products quickly from one-pot reactions, leading to more sustainable chemical syntheses.
